# A High-Density EEG Study Investigating the Neural Correlates of Continuity Editing Theory in VR Films

**DOI:** 10.3390/s23135886

**Published:** 2023-06-25

**Authors:** Wanqiu Cheng, Xuefei Wang, Jiahui Zou, Mingxuan Li, Feng Tian

**Affiliations:** 1 Shanghai Film Academy, Shanghai University, Shanghai 200072, China; 2 Shanghai Film Special Effects Engineering Technology Research Center, Shanghai University, Shanghai 200072, China

**Keywords:** electroencephalogram (EEG), virtual reality (VR) film, attentional theory of continuity editing, visual evoked potential

## Abstract

This paper presents a cognitive psychology experiment to explore the differences between 2D and virtual reality (VR) film editing techniques. We recruited sixteen volunteers to view a range of different display modes and edit types of experimental material. An electroencephalogram (EEG) was recorded simultaneously while the participants watched. Subjective results showed that the VR mode reflects higher load scores, particularly in the effort dimension. Different editing types have no effect on subjective immersion scores. The VR mode elicited stronger EEG energy, with differences concentrated in the occipital, parietal, and central regions. On the basis of this, visual evoked potential (VEP) analyses were conducted, and the results indicated that VR mode triggered greater spatial attention, while editing in 2D mode induced stronger semantic updating and active understanding. Furthermore, we found that while the effect of different edit types in both display modes is similar, cross-axis editing triggered greater cognitive violations than continuity editing, which could serve as scientific theoretical support for the development of future VR film editing techniques.

## 1. Introduction

**Figure 1:**
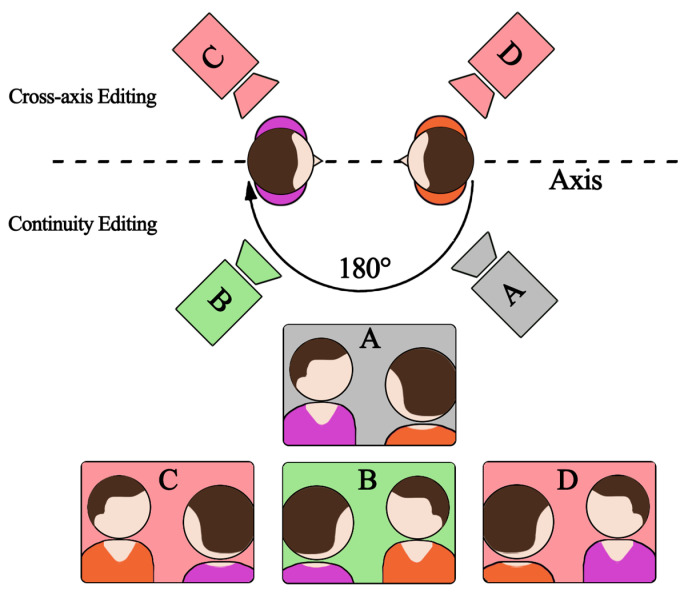
Showing the 180-degree rule in continuity editing. Camera A is the establishing shot, camera B is the continuity editing shot, and cameras C and D are the cross-axis editing shots.

As one of the mainstream mediums of recording and narrative expression, film has evolved and innovated over the centuries, and nowadays, films rarely consist of a single shot but are composed of many fragments combined and stitched together according to certain rules to form a completed story. The clips are shot from different angles of camera placement, but the audience is still able to understand the director’s intention to translate this not-quite-continuous information into a coherent event. Film artists attribute this to an invisible continuity editing system [1], which is a traditional narrative film presentation of a dramatic plot that ensures temporal and spatial unity, while the editor needs to anticipate and gently control the audience’s mind, not allowing them to feel the presence of the director, so that the audience can watch in an “unconscious” and natural state. This editing system is still widely used today. For the shot editing of dialogue scenes, the audience needs to understand the relationship between the actors’ positions. Continuity editing follows a basic scheduling rule, the “axis rule”, or the 180-degree rule [2]. The so-called axis refers to a virtual straight line formed between the subject’s direction of vision, the direction of motion, and the object of communication with it. In actual shooting, the camera is positioned within a 180-degree area to ensure a consistent sense of direction and space in each shot. As shown in Figure 1, the director establishes this axis by introducing the scene through an “establishing shot” (camera A), and each subsequent shot must be taken from the same side of the axis (camera B). If the camera crosses the axis (cameras C and D), the left-right relationship on the screen will be reversed (see the difference between B and C). This violation of the 180-degree rule is called “cross-axis” and may affect the viewer’s understanding of the film.

The virtual reality (VR) film industry is gradually emerging, with prestigious film festivals like Cannes, Venice, Tribeca, and Sundance holding out olive branches to virtual reality films and setting up virtual reality sections one after another. As a new form of film art creation, it can give audiences an aesthetic and cinematic experience of spectacle. Compared with traditional films, virtual reality films have a stronger sense of immersion and interactive features, which inject new momentum into the development of the film and television industry. How the visual language and expression of virtual reality films differ from traditional films is a question that all virtual reality video artists are thinking about. Since virtual reality films have essential narrative differences from traditional films, some film creators believe that it is difficult to form a non-linear and complex combination of montages in virtual reality, which is more suitable for simple transitions [3]. That is, a large number of fixed shots are used, articulated only by jump cuts when scenes change, thus avoiding triggering dizziness and physical contradictions in the viewer. More film creators believe that editing is equally important in virtual reality films, and as an emerging film art form, a set of art theory systems based on the characteristics of virtual reality films should be explored based on the aesthetic experience requirements of virtual reality films. Some of the existing research focuses on the practical application of theory, integrating virtual reality film directing techniques from a practical perspective [4,5], while another part explores VR film editing techniques and cognitive theory from a more objective perspective. Kjær et al. [6] proposed that appropriate editing can direct the viewer’s attention. Serrano et al. [7] discussed the relationship between film editing and cognitive event segmentation in VR films, the first to provide a systematic analysis of viewer behavior and the perception of continuity in VR. In a study by Balzarotti et al. [8], it was confirmed that editing density affects viewers’ perception of time and that edited clips are more likely to trigger eye movements. Rothe et al. [9] explored and evaluated the classification of methods to guide user attention classification in VR films, combined with eye-tracking methods to explore the interaction techniques of VR films, and analyzed the production methods of nonlinear interactive VR films.

Neurocinematography, as the intersection of neuroscience and cinematography, aims to use cognitive neuroscience approaches to explore the mechanisms of film viewing and the relationship between audiences and films. The idea that films can influence the minds of viewers has been proposed since the early days of the film industry, but it was not until the advent of non-invasive neuroimaging technology in the early 1990s that it became possible to observe and record the minds and neurological states of viewers during film viewing. Since Hasson’s team first introduced the concept of neurocinematography in 2008, many scholars have used it to study classical film theory, and neurocinematography has become a new direction for studying and expanding the development of film theory [10]. Current studies usually use electrocardiograms (ECGs) [11,12], functional magnetic resonance imaging (fMRI) [13], and electroencephalograms (EEGs) [14] as assessment metrics. The EEG stands as one of the earliest techniques for measuring neuronal activity within the human brain [15], with ultra-high temporal resolution. As a non-invasive technology, an EEG enables the recording of cerebral evoked potentials from the surface of the skull. These recordings reflect neurophysiological changes occurring in the brain during cognitive processes, including attention, perception, emotion, movement, decision making, and judgment, providing a reliable basis for studying information processing [16]. In addition, significant voltage fluctuations caused by evoked brain activity are known as event-related potentials (ERPs). Mental processes, such as selective attention, perception, memory, and language processing, occur over a period of tens of milliseconds [17]. ERPs are used to define the temporal evolution of these activations [18]; there are some frequently discussed ERPs in visual tasks. The P300 can reflect neurophysiological changes in the brain during cognition processes [19] and is triggered when stimulus detection is involved in memory manipulation, responding to the cognitive demands during task processing. The N400 has now been used to detect semantic or structural violations at the level of music, visual scenes, actions, etc. [20,21,22]. For traditional 2D films, it has been found that film editing induces certain physiological responses, such as lower heart rate [23] and lower pulse rate [16]. Andreu-Sánchez et al. [24] analyzed the effects of media specialization on cognitive neurodynamics during audiovisual cutting using EEG data. Rosenfeld et al. [25] make the point that for films of different levels of interest, there is no difference between N100 and P300 for the viewer. Other studies have explored the EEG components associated with editing, demonstrating that editing triggers early ERP components and that different editing mechanisms trigger different EEG components [26]; when processing audiovisual messages, the brain distinguishes between related and unrelated cuts [27], unrelated cuts can trigger the P300 components [28], and the N400 could be detected in the frontal, parietal, and central regions in film editing [29]. VR environments result in greater early visual attention and higher α power than 2D films [30]. Tian et al. [31] studied VR film editing and cognitive event segmentation theory using high-density EEGs, concluding that spatial changes had the greatest impact on viewers and that editing could help better understand the footage. Cao et al. [32] conducted a preliminary exploration of the implementation of montage editing techniques in VR films, arguing that the VR portal effect may benefit the viewer’s cognitive load compared to clipping and fading, but may negatively affect recall performance. However, research into how different editing in different display modes evoke cognition and load is still at a preliminary stage.

In this paper, we investigate the brain-neural mechanisms of viewers’ stimulus presentation of film clips with different editing methods under two different viewing conditions, traditional 2D planes and VR environments, combine subjective evaluation, explore the usability of traditional film continuity editing methods in VR films, and broaden VR film editing techniques and creation theories.

In general, we focus on the following key issues:

- What are the electrophysiological differences between watching a traditional film and a VR film?
- Are there electrophysiological differences between watching continuity clips and cross-axis clips?

The subsequent sections of this paper are organized as follows. Section 2 presents the details of the experimental contents and procedures, data recording methods, and the pre-processing of EEG data. Section 3 describes the analytical process and results of the experiment in detail. The results of the experiment are discussed in Section 4. Lastly, the conclusions of the experiment are presented in Section 5.

## 2. Materials and Methods

In order to minimize experimental error, we conducted a pre-experiment before the start of the formal experiment. The data from the formal experiment consisted of two components: a subjective measure and an objective EEG signal. The subjective scale questionnaires are the NASA Task Load Index (NASA-TLX) [33] and the Immersion Questionnaire (IPQ) [34], and the objective EEG data are EEG experiments based on visual stimuli with different display modes and editing types. The variables studied were assumed to affect the viewer’s load, immersion, perception, and enjoyment of the experimental materials, but not the actual viewing.

### 2.1. The Participants

For this experiment, a total of 16 participants (9 males and 7 females) with an average age of 23.25 years were recruited. We ignore the effect of gender in this experiment because there is no literature that mentions that the gender of normal adults affects the content of this study. All participants in the experiment were students at Shanghai University, with right-handedness as the dominant hand, and normal or corrected vision. Moreover, none of the participants had a history of mental illness. Participants were randomized to complete two sets of experimental data collection in different viewing modes over two separate days, with each experiment taking approximately 1.5 h, thus avoiding fatigue effects from prolonged experiments. All participants completed self-assessment questionnaires (Anxiety Self-Rating Inventory and Depression Self-Rating Inventory) prior to the start of the experiment, which showed that the participants had a normal recent psychological profile with no tendency towards anxiety or depression to ensure the validity of subsequent experimental data. We set aside 15 min for participants to familiarize themselves with the experimental environment and explained to them the basic operations and precautions to be taken during the experiment, after which they were guided to watch clips of material from the formal experiment, and they were required to fill in subjective questionnaires (NASA-TLX and IPQ) after viewing each set of experimental material. The experiment was approved by the Science and Technology Ethics Committee of Shanghai University. All participants participated voluntarily, signed an informed consent form before the experiment, and were paid accordingly at the end of the experiment.

### 2.2. Experimental Materials and Hardware Equipment

**Figure 2:**
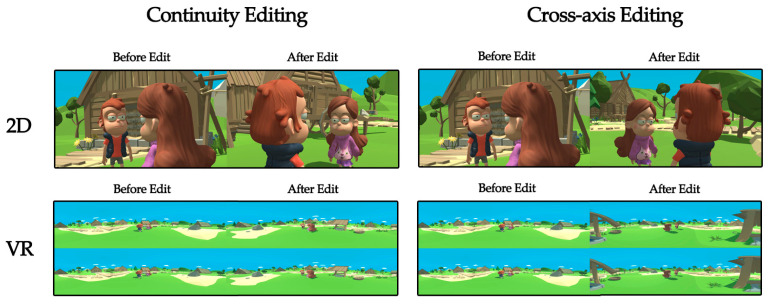
Shown are clips of the experimental materials in different conditions. There are two display modes (2D or VR) and two edit types (continuity editing and cross-axis editing). Under each edit type, the left side is before the edit and the right side is after the edit.

This study is concerned with the design of the camera language of a two-person dialogue film. In response to these issues, the experimental stimulus is set up by the 180-degree rule in the continuity editing technique in two dimensions: display mode and edit type. The display mode is VR or 2D, and the editing type is continuity editing or cross-axis editing. Specifically, each mode contains 8 sets of experimental clips, 4 of which are continuity editing clips and 4 are cross-axis editing clips. The continuity clips and the cross-axis clips have the same content, only the direction of the axis of the shot is changed. Each set of experimental footage contains 25 clips, each are 5 s in length. Therefore, the experimental material for the VR group contains 8 groups of clips from the VR condition, each with 25 videos, for a total of 200 stimuli. Similarly, the 2D group changes only the viewing mode and consists of the same 8 sets of 200 segments of the experimental material. The material for all the experiments was animated using Maya 2018, with camera placement and parallax restored in Unity 2018.4, edited and composited using Adobe After Effects CC2018, with a video format encoded in H.264 at 4096 × 2048 resolution and a frame rate of 30 frames per second. The experimental materials for different conditions are shown in Figure 2.

In both display conditions, the same PC was used, consisting of a 3.4 GHz Intel Xeon E5-1230 V5 processor, 32 GB RAM, and an NVIDIA GTX 1070D. The PC connected to an AOC 24-inch LCD monitor, while the HMD headset monitor was an HTC VIVE.

### 2.3. Experimental Procedure

**Figure 3:**
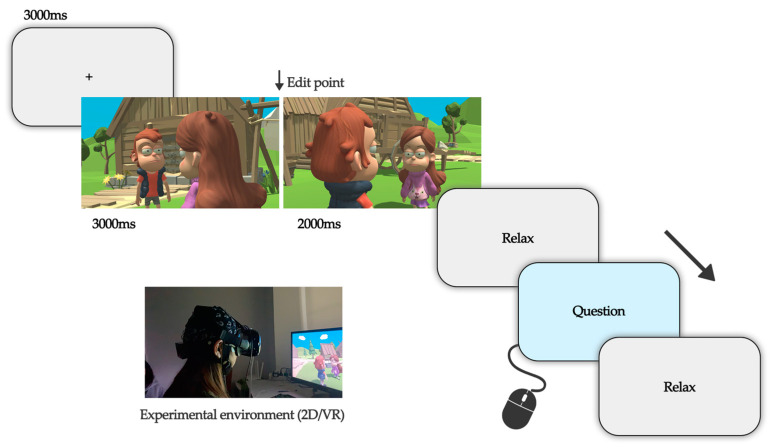
Experimental flow and experimental environment.

The experiments were conducted in a closed and soundproof environment, with all communication devices switched off and placed outside the laboratory. The experimenter assists the participant to sit in a suitable position in front of the display, calibrates each participant while viewing with the VR device, and controls the distance between the participant and the screen while viewing in 2D mode. During the EEG signal acquisition, 64-channel electrode caps were used, and electrode placement followed the international 10–20 system. Each subject washed their hair at least 2 h prior to the formal experiment to keep the scalp dry and clean. The experimenter injects EEG paste on the subject’s scalp at the location of the corresponding electrodes to reduce the impedance and simultaneously observes the impedance state of each EEG channel in the monitoring software, and when the impedance of all channels is reduced to below the standard threshold (5 kΩ), the EEG signal acquisition equipment is considered to be completed, and the signal transmission of each acquisition channel on the EEG cap is always observed to be normal during the experiment. To reduce noise interference in the acquisition of EEG signals, subjects are advised not to speak, make forceful facial expressions, blink frequently, or shake their limbs significantly during viewing. To ensure that the experiment runs smoothly, we use other short video clips to pre-experiment and inform the subjects about the procedure and operation of the experiment, after which we proceeded to the formal content of the experiment. For the same subject, experiments in both 2D and VR modes were completed over two days, the order in which the two modes are viewed is randomized, as is the order in which the experimental material is played for each group. Before the formal viewing of the footage, we collected resting-state EEG signals from subjects in a closed-eye state for 3 min and then began the formal viewing of the footage through a 2D screen or VR equipment. At the end of each set of experimental clips viewed, subjects fill in a subjective test scale, after which participants can take a break at their own discretion to reduce the fatigue effect, as shown in Figure 3 in the experimental flow.

### 2.4. Data Recording and Processing

The subjective data for this paper was collected by means of an electronic questionnaire, which the subject filled out in the questionnaire interface, according to the feelings of each group of experiments, and clicked submit to complete the subjective data collection for that subject. The experiment was analyzed using the NASA-TLX and IPQ questionnaires to calculate each subject’s score, then the data was restructured according to the subscales analyzed, edited in an Excel spreadsheet, and imported into SPSS software for analysis from different perspectives.

For EEG data recording and pre-processing, this paper uses Neuracle EEG Recorder V2.0.1 for EEG data recording, the reference electrode is Cpz, the ground electrode is AFz, the sampling frequency of the EEG detector is set to 1000 Hz, and the electrode impedance is less than 5 kΩ during signal a recording. The EEG data monitoring software records the participants’ EEG signals simultaneously and is monitored by the experimenter in real time to ensure the signal is collected and recorded correctly. Pre-processing of EEG data is carried out with the EEGLab toolbox. In order to eliminate industrial frequency interference, the raw data were filtered in the pre-processing step with a bandpass filter of 0.1–90 Hz, followed by a trap filter of 50 Hz and 100 Hz [35]. We observed the waveform of the EEG data, removed data segments where the overall waveform is heavily skewed, and if only individual channels of the data segments have significant drift in the waveform, interpolation is performed. After rejecting defective segments and interpolating the bad leads, we applied independent component analysis (ICA) in EEGLab to get away from the artifact components [36] and carried out manual removal of artifacts such as eye movements, eye drift, myoelectricity, etc. According to our experimental task, the EEG data prior to the stimulus onset was chosen as the baseline for each stimulus segment [14] and re-referenced.

For the EEG signals, after the above pre-processing work, the EEG signal is divided into frequencies using a band-pass filter. EEG signals in the α band (8–13 Hz), β band (13–18 Hz), and θ band (4–7 Hz) were selected for subsequent specific analysis. During the analysis, feature channels were extracted separately for the following brain regions: frontal region (Fz, F3, F4, FCz, FC3, and FC4), central region (Cz, C3, C4, CP3, and CP4), parietal region (Pz, P3, and P4), temporal region (TP7, TP8, T7, T8, P7, and P8) and occipital region (POz, PO3, PO4, Oz, PO7, and PO8) [37]. Extraction of the average energy of the 25 test segments of the EEG signal corresponding to each channel and the energy of the data segment within a frequency band was represented as the logarithm of the sum of the squares of all data points within that band in base of 10, as shown in Equation (1), where k represents the number of trials in the data segment, n represents the number of data points in each segment, and $x(k{)}_{i}$ represents the value of the ith point in the kth data segment.

(1) $$E(k)=\mathrm{lg}\left[\sum_{i=1}^{n}x(k{{)}_{i}}^{2}\right],$$

For the VEP data, the 0.1–90 Hz filter range in the above pre-processing work was used to check whether the high-frequency information of the EEG data was retained intact. The EEG data of subjects with incomplete retention of high-frequency information were not subjected to subsequent analysis, and the actual filter range for the analysis was 0.1–30 Hz. During the analysis, the same feature channels were selected for the EEG statistical analysis, focusing on data from the frontal region (Fz, F3, F4, FCz, FC3, and FC4) and occipital region (POz, PO3, PO4, Oz, PO7, and PO8). In order to find the ERP components triggered by the two different editing types, we chose 1000 ms before the appearance of the edit point in the experimental material as the baseline for each segment of data and analyzed the ERP components within 1000 ms after the appearance of the edit point stimulus. Selection of time windows for analysis is based on previous literature. A total of four time windows were chosen to analyze ERP amplitudes on the scalp surface [26]: time window 1, 140–190 ms after stimulus onset; time window 2, 180–220 ms after stimulus onset; time window 3, 250–380 ms after stimulus onset; and time window 4, 400–650 ms after stimulus onset.

## 3. Results

In this paper, a multi-way ANOVA was used to explore the interaction of display mode × edit type × region for the EEG data. A two-way ANOVA was used to explore the interaction of display mode × edit type for the subjective data from the NASA-TLX table and the VEP data. A one-way ANOVA was used on subjective data from the IPQ table to explore the effect of edit type. In the ANOVA, if there were interactions between the factors, a simple effects analysis was performed and corrected for multiple comparisons using the Bonferroni method. All analyses were performed with *p* < 0.05 as a measure of significance and statistics were performed using SPSS 26.0 (IBM, Armonk, NY, USA).

### 3.1. Subjective Data

The NASA-TLX scale evaluates the participant’s perceived load in six dimensions: mental demand (MD), physical demand (PD), temporal demand (TD), effort (E), performance (P), and frustration level (FL). The IPQ scale consists of three dimensions: spatial presence (SP), involvement (INV), and reality (REAL). The level of the score reflects the level of load and immersion. For the subjective data, the subscale scores and total scores of the NASA-TLX and IPQ subjective questionnaires were averaged and analyzed for all volunteers, and the statistical results of the total scores of the two scales are shown in Table 1.

**Figure 4:**
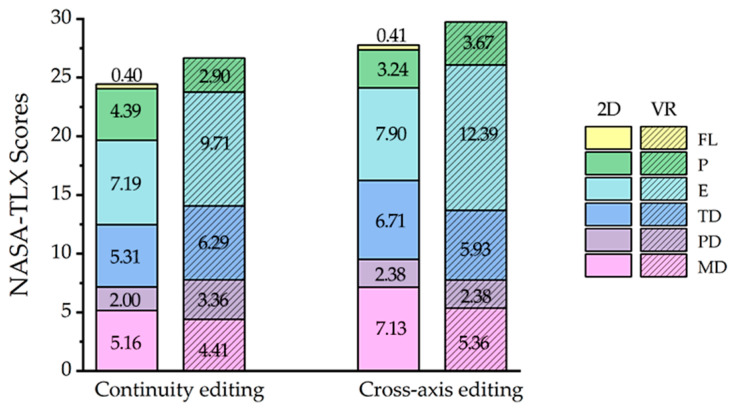
Subscale score statistics for the NASA-TLX scale at the display mode and edit type grouping level. MD: mental demand, PD: physical demand, TD: temporal demand, E: effort, P: performance, and FL: frustration level.

In the NASA-TLX scale, we perform a 2 × 2 ANOVA (two display modes × two edit types) on the six subscale scores (MD, PD, TD, E, P, and SP) scores and the total scale scores, with the following results: for the effort subscale, mode (F(1, 256) = 11.519, *p* = 0.001 < 0.05, η^2^ = 0.076) has a significant main effect, VR mode shows a higher effort score than 2D mode; for the frustration level subscale, mode (F(1, 256) = 11.277, *p* = 0.001 < 0.05, η^2^ = 0.075) has a significant main effect, VR mode shows a lower frustration level score than 2D mode. The effect of the edit type factor on the scores in the NASA-TLX scale was not statistically significant, but the cross-axis editing clips had higher load scores compared to the continuity editing clips. The subscale scores for NASA-TLX under the display mode and edit type grouping level are shown in Figure 4. In the IPQ scale, there were no significant differences in subscale scores and total scale scores when participants viewed the two edit types of experimental materials in VR mode.

### 3.2. EEG Data Analysis

**Figure 5:**
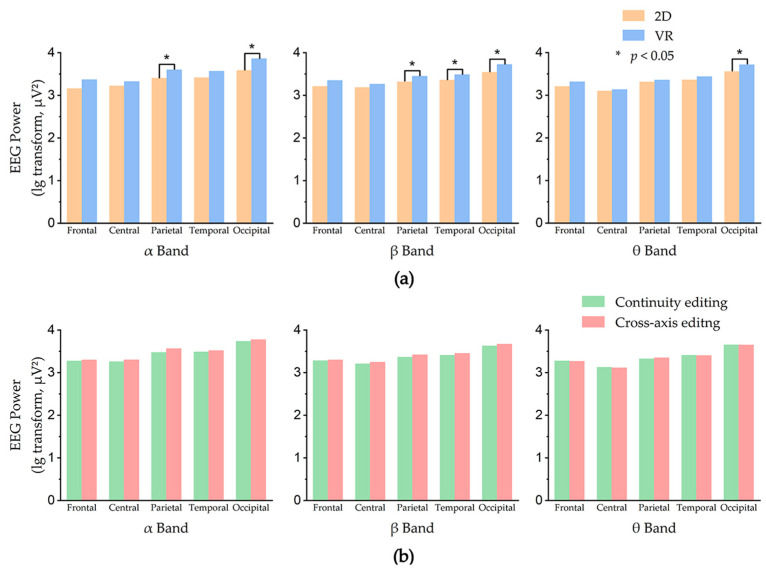
EEG power in the α, β, and θ bands of the different regions. (**a**) EEG power in 2D and VR modes. (**b**) EEG power in continuity editing and cross-axis editing.

This section analyzed EEG power from α, β, and θ bands separately. We used a multi-way ANOVA to explore the interaction of display mode × edit type × region. The EEG power of each region in different display modes and edit types is shown in Figure 5.

#### 3.2.1. α Band

A 2 × 2 × 5 ANOVA (two display modes × two edit types × five regions) was performed for the α band, and the results were as follows: mode × region (F(4, 875) = 4.140, *p* = 0.003 < 0.05, η^2^ = 0.019) had significant interaction. The simple individual effects analysis suggested that for different display modes, the α band power in different regions showed significant differences: 2D (F(4, 875) = 5.177, *p* < 0.001, η^2^ = 0.023); VR (F(4, 875) = 29.099, *p* < 0.001, η^2^ = 0.119). Paired comparison results showed, in the 2D mode, the α band power in the occipital region was significantly higher than the frontal region (*p* < 0.001) and central region (*p* = 0.002); in the VR mode, the α band power in the occipital region was significantly higher than other regions (*p* < 0.001). For different regions, the α band power was higher in VR mode than in 2D mode and showed significant differences in the parietal region (*p* = 0.010) and occipital region (*p* < 0.001). The effect of the edit type factor on the EEG signal in the α band was not statistically significant. In all regions, α band power was higher in the cross-axis editing than in the continuity editing clips.

#### 3.2.2. β Band

A 2 × 2 × 5 ANOVA (two display modes × two edit types × five regions) was performed for the β band, and the results were as follows: mode × region (F(4, 875) = 3.385, *p* = 0.009 < 0.05, η^2^ = 0.016) had significant interaction. The simple individual effects analysis suggested that for different display modes, the β band power in different regions showed significant differences: 2D (F(4, 875) = 11.475, *p* < 0.001, η^2^ = 0.050); VR (F(4, 875) = 40.761, *p* < 0.001, η^2^ = 0.159). Paired comparison results showed, in the 2D mode, the β band power in the occipital region was significantly higher than the parietal region (*p* < 0.001), frontal region (*p* < 0.001), temporal region (*p* = 0.003), and central region (*p* < 0.001); in the VR mode, the β band power in the occipital region was significantly higher than other regions (*p* < 0.001). For different regions, the β band power was higher in VR mode than in 2D mode and showed significant differences in the parietal region (*p* = 0.021), temporal region (*p* = 0.014), and occipital region (*p* < 0.001). The effect of the edit type factor on the EEG signal in the β band was not statistically significant. In all regions, β band power was higher in the cross-axis editing than in the continuity editing clips.

#### 3.2.3. θ Band

A 2 × 2 × 5 ANOVA (two display modes × two edit types × five regions) was performed for the θ band, and the results were as follows: mode × region (F(4, 875) = 3.888, *p* = 0.004 < 0.05, η^2^ = 0.018) had significant interaction. The simple individual effects analysis suggested that for different display modes, the θ band power in different regions showed significant differences: 2D (F(4, 875) = 16.541, *p* < 0.001, η^2^ = 0.071); VR (F(4, 875) = 52.008, *p* < 0.001, η^2^ = 0.194). Paired comparison results showed, in both 2D and VR modes, the θ band power in the occipital region was significantly higher than in other regions (*p* < 0.001). For different regions, the θ band power was higher in VR mode than in 2D mode and showed significant differences in the occipital region (*p* < 0.001). The effect of the edit type factor on the EEG signal in the θ band was not statistically significant. In all regions, θ band power was similar in the continuity editing and cross-axis editing clips.

### 3.3. Brain Topography

**Figure 6:**
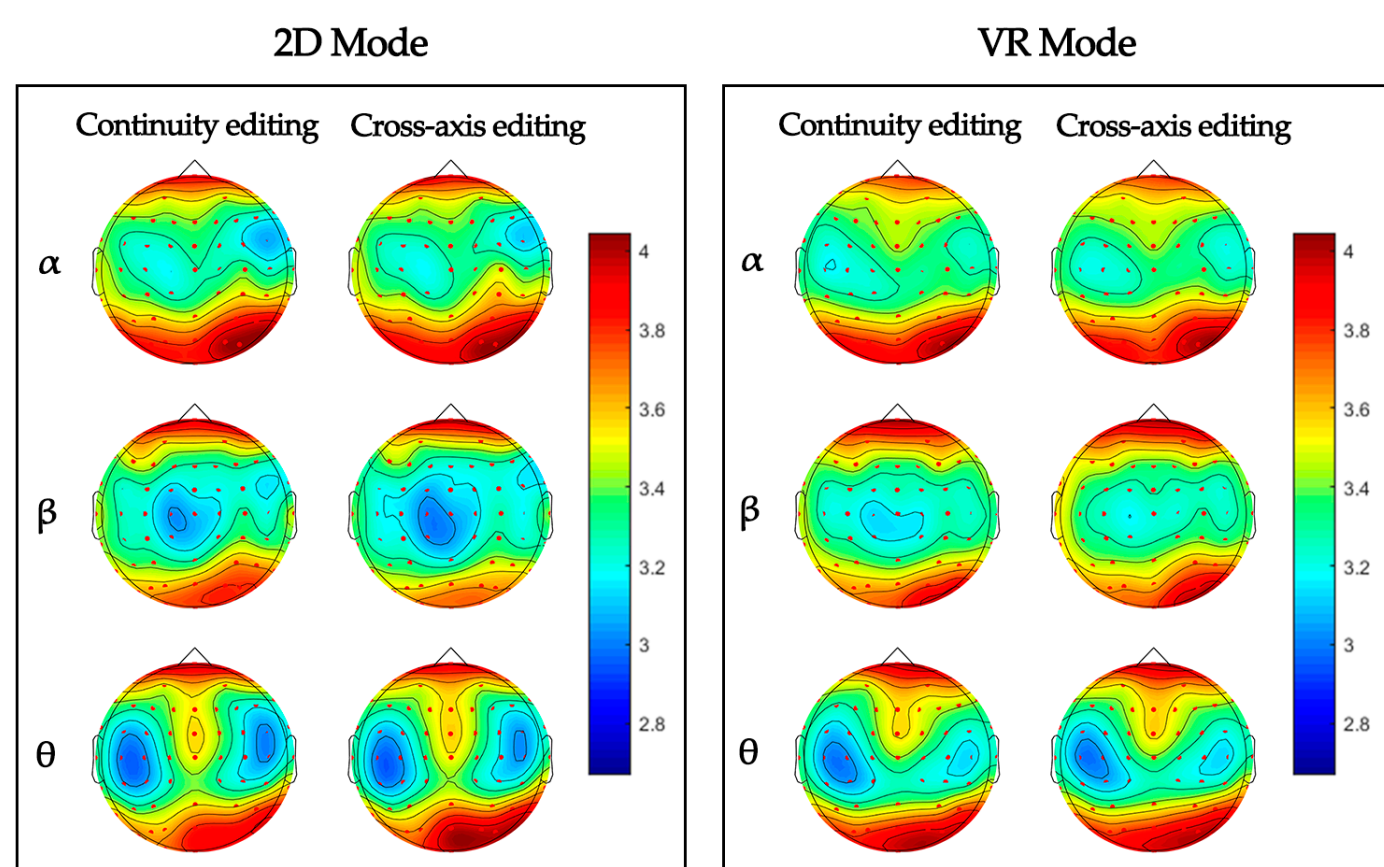
Brain topography in the α, β, and θ bands evoked by different display modes and edit types. The brain topography showed the results of averaging the brainwave energy of all participants in each condition.

Warm tones indicate higher brainwave activity and cold tones indicate lower brainwave activity in Figure 6. VR mode has warmer brain topography than 2D mode.

### 3.4. VEP Data Analysis

**Figure 7:**
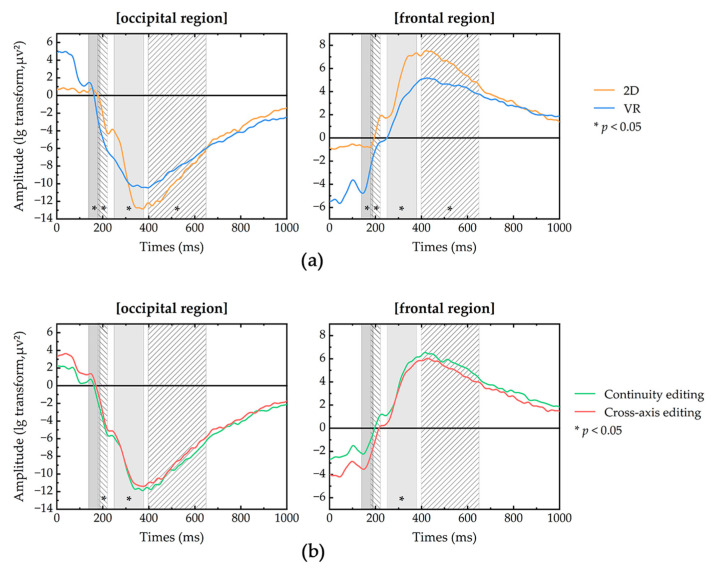
Differences in VEP amplitudes between occipital and frontal regions at the mode and edit factor levels. (**a**) 2 display modes, (**b**) 2 edit types.

In this section, for each of the four selected time windows, we used a two-way ANOVA to explore the interaction of display mode × edit type. The main results of the VEP are shown in Figure 7.

In the first time window (140–190 ms), a 2 × 2 ANOVA (two display modes × two edit types) was performed on the VEP data in the occipital and frontal regions individually. The results showed, for the occipital and frontal regions, there was a significant main effect of mode. For the occipital region, mode (F(1, 176) = 43.101, *p* < 0.001, η^2^ = 0.200); for the frontal region, mode (F(1, 176) = 59.895, *p* < 0.001, η^2^ = 0.258). However, for the occipital region, the 2D mode evoked lower power than the VR mode, and in the frontal region, the results were reversed, with the 2D mode evoking higher power than the VR mode. The effect of the edit type factor on the VEP in the occipital and frontal regions was not statistically significant.

In the second time window (180–220 ms), a 2 × 2 ANOVA (two display modes × two edit types) was performed on the VEP data in the occipital and frontal regions individually. The results showed, for the occipital and frontal regions, there was a significant main effect of mode. For the occipital region, mode (F(1, 176) = 22.338, *p* < 0.001, η^2^ = 0.115); for the frontal region, mode (F(1, 176) = 49.273, *p* < 0.001, η^2^ = 0.223). For the occipital region, the 2D mode evoked lower power than the VR mode, and in the frontal region, the 2D mode evoked higher power than the VR mode. Edit type (F(1, 176) = 5.512, *p* = 0.020 < 0.05, η^2^ = 0.031) had a significant main effect in the occipital region; continuity editing evoked significantly lower power than cross-axis editing.

In the third time window (250–380 ms), a 2 × 2 ANOVA (two display modes × two edit types) was performed on the VEP data in the occipital and frontal regions individually. The results showed significant main effects of both mode and edit in the occipital and frontal regions. For the occipital region, mode (F(1, 176) = 17.591, *p* < 0.001, η^2^ = 0.093), and edit (F(1, 176) = 11.157, *p* = 0.001 < 0.05, η^2^ = 0.061). For the frontal region, mode (F(1, 176) = 20.596, *p* < 0.001, η^2^ = 0.107), and edit (F(1, 176) = 6.306, *p* = 0.013 < 0.05, η^2^ = 0.035). For the occipital region, the 2D mode evoked lower power than the VR mode, and the continuity editing evoked significantly lower power than cross-axis editing. For the frontal region, the 2D mode evoked higher power than the VR mode, and the continuity editing evoked significantly higher power than cross-axis editing.

In the fourth time window (400–650 ms), a 2 × 2 ANOVA (two display modes × two edit types) was performed on the VEP data in the occipital and frontal regions individually. The results showed, for the occipital and frontal regions, there was a significant main effect of mode. For the occipital region, mode (F(1, 176) = 8.767, *p* = 0.004 < 0.05, η^2^ = 0.049); for the frontal region, mode (F(1, 176) = 7.537, *p* = 0.007 < 0.05, η^2^ = 0.042). For the occipital region, the 2D mode evoked lower power than the VR mode, and in the frontal region, the 2D mode evoked higher power than the VR mode. The effect of the edit type factor on the VEP in the occipital and frontal regions was not statistically significant.

## 4. Discussion

Our work investigates the neurophysiological effects of continuity editing techniques in VR films, revealing some potential possibilities for applying traditional continuity editing techniques in VR. In this section, we first summarize our findings and present our insights, and then discuss the problems we faced in our experiments, which we hope will shed light on future work in the field.

### 4.1. Subjective Rating

In the subjective data, for NASA-TLX scale results, we found different load scores on the NASA-TLX scale for the different display modes, with higher load scores for the VR mode than for the 2D mode and significant differences in the subscales of effort. This may be due to the greater immersion and information brought about by the VR environment, with participants needing to exert more effort to process the content of the film clips. For the different edit types of 2D videos, Kachkovski et al. [2] proved that cross-axis editing can lead to confusion and disorientation for viewers, but does not affect the viewers’ enjoyment. The results of the NASA-TLX scale showed that this conclusion applies not only in 2D display mode but also in VR display mode; for the performance subscale, scores were similar between edit types. It can be presumed that the different edit types do not affect the subjective satisfaction of the viewers. The effect of edit types on load was not statistically different in scores on the total scale but showed a trend of higher scores for the cross-axis editing than the continuity editing clips, which may be due to the small size of our statistics and sample size that could be increased in future work to inform further research on the effect of editing methods on viewing load. For IPQ scale results, Tian et al. [31] demonstrated that there is a difference in total IPQ scores between editing and no editing, but no statistical difference in IPQ scores between different editing groups. Our work further discussed the differences in IPQ total and subscale scores by edit type, and the results showed no statistical differences between edit type on both total and subscale scores; therefore, we hypothesized that edit type is not a major factor affecting participants’ subjective immersion.

### 4.2. EEG Results

The cerebral cortex is in charge of the cognitive and emotional functions of the brain and is divided into several brain regions according to their different functions. The frontal lobe is responsible for thinking, planning, and central executive functions as well as motor execution; the parietal lobe is responsible for somatosensory perception and the integration of visual and spatial information; the temporal lobe is mainly responsible for language functions and auditory perception and is involved in emotion and long-term memory; and the occipital lobe is primarily responsible for the perception and processing of visual information. Research in neuroscience has shown that neural oscillations have an important role to play in human cognition. The α waves have been shown to be associated with cognitive activity in humans, which may include attentional load, cognitive arousal, or mental effort [38,39]. β waves reflect emotions in the brain and are associated with cognitive effort, and they increase significantly in energy during more complex tasks [40,41]. θ waves in the frontal lobe increase during a variety of working and situational memory tasks [42].

The experimental results showed there is no interaction effect between display modes and editing types. In different display modes, the VR mode evoked stronger brain activity than the 2D mode in all the frequency bands analyzed, and there were significant differences in the occipital region where visual perception and processing occurred, as well as significant differences in the amplitudes of the α and β bands in the parietal region and the β band in the temporal region in both display modes. From this, it can be concluded that the VR display mode is a more complex visual cognitive task compared to the 2D display mode, evoking stronger attentional load, cognitive arousal, and mental effort. This is consistent with the results of the NASA-TLX subjective scale. In different edit types, the average power in α and β bands was higher for cross-axis editing clips than for continuity editing clips, but our results do not reflect a significant difference, which may be due to our smaller statistics.

Overall, VR mode had greater immersion and information and triggered more intense brain activity relative to the 2D mode, which we hypothesize is a form of attentional load, cognitive arousal, or mental effort. In different editing types, cross-axis editing may present more complex cognitive tasks that induce more intense brain activity compared to continuity editing clips.

### 4.3. VEP Results

VEP is an evoked electrophysiological potential that can provide important diagnostic information about the functional integrity of the visual system [43], and it reflects the conduction function of the retina to the primary visual cortex. P100 is associated with the attention elicited by visual tasks, is influenced by stimulus contrast, and is sensitive to the direction of spatial attention [44]. P300 reflects the neurophysiological changes in the brain during cognition and is triggered when stimulus detection is involved in memory manipulation, responding to the cognitive demands during task processing. P300 reflects the neurophysiological changes in the brain during cognition and is triggered when stimuli are detected to be involved in memory operations. It has been shown that different editing techniques have different effects on the viewer and that related cuts can trigger a large number of negative potentials [28]. N400 is related to the treatment of meaning; it has a greater amplitude when the new element does not match the context established by the previous stimulus [45].

The experimental results showed there is no interaction effect between display modes and editing types. The amplitudes evoked by the different display modes were statistically different in both the occipital and frontal regions during the four time windows. In the first time window, the change in P100 amplitude triggered by the VR mode is greater, suggesting that stronger spatial attention is required in VR films. In the second and third time windows of the frontal region, the 2D mode elicited relatively larger amplitudes and shorter latencies, and the VR display mode had a longer P300 latency. The degree of difficulty of the task can affect latency and wave amplitude changes, with the greater the difficulty, the more pronounced the P300 potential; it can be hypothesized that the immersive environment of VR mode may help the viewer’s understanding of the edited clips. In both display modes, the clip produced a large negative potential in the occipital region, which started at 100 ms and extended beyond 800 ms. This is consistent with the findings of Matran-Fernandez et al. [28] and stems from the need for subjective attentional regulation to update and integrate semantic information after editing. The N400 amplitude was triggered later and more strongly in the 2D mode than in the VR mode; it can be speculated that the viewers had a stronger semantic update and active understanding of the 2D films. The amplitudes evoked by the different edit types were statistically different in both the occipital and frontal regions during the four time windows. The VEP amplitudes evoked by different edit types were statistically different in the second and third time windows in the occipital region and in the third time window in the frontal region. Cross-axis editing evoked greater variation in P300 amplitude, which may have triggered cognitive violations, a more difficult task compared to continuity editing. In the fourth time window, there was no statistical difference in the VEP amplitude induced by the two edit types, which may be the result of autoregulatory inhibition in the brain.

Overall, the VR mode required stronger spatial attention, but the immersive environment of the VR mode may contribute to viewers’ understanding of the content of the film clips. After the editing of the clips, viewers need to regulate their attention and integrate new semantic information, and the cross-axis editing triggered greater cognitive violations and showed the same trend in both display modes.

## 5. Conclusions

VR films have a different level of immersion and cognitive experience due to VR being a different viewing medium than traditional films. While traditional film theory is flourishing, the use of cognitive neuroscience to explore VR film theory has become a scientifically valid research trend. This study focuses on exploring the subjective and objective effects of different display modes and editing types on viewers based on traditional film theory through subjective ratings, EEG energy features, and VEP.

Overall, our study confirms the scientific validity of traditional film theory in 2D films, cross-axis editing triggers a greater load than continuity editing, with the same trend in both display modes. From this, it can be concluded that the 180-degree rule of traditional film continuity editing techniques can also be used in VR films. VR mode triggers a greater effort load and spatial attention; editing in 2D mode induced stronger semantic updating and active understanding, and cross-axis editing is a risky editing technique compared to continuity editing. These findings can be used as scientific theoretical support for the development of future VR film editing techniques. However, due to the small number of participants and the small power of the statistics, for future studies we should include more participants and richer experimental materials in order to obtain more homogeneous and richer experimental results.

## Figures and Tables

**Table 1:** The average value (mean ± S.D.) of total scores in NASA-TLX and IPQ (16 participants).

Display Mode	Edit Type	NASA-TLX Scores	IPQ Scores
2D	Continuity editing	24.45 ± 12.66	/
	Cross-axis editing	27.77 ± 13.58	/
VR	Continuity editing	26.67 ± 14.10	−4.03 ± 8.73
	Cross-axis editing	29.73 ± 17.06	−3.51 ± 11.06

## Data Availability

The data presented in this study are available on request from the corresponding author. The data are not publicly available because we are creating an EEG data set.
